# Miconazole induces aneuploidy-mediated tolerance in *Candida albicans* that is dependent on Hsp90 and calcineurin

**DOI:** 10.3389/fcimb.2024.1392564

**Published:** 2024-06-25

**Authors:** Liangsheng Guo, Lijun Zheng, Yubo Dong, Chen Wang, Huijie Deng, Zongjie Wang, Yi Xu

**Affiliations:** ^1^ Department of Obstetrics and Gynecology, The Second Affiliated Hospital of Soochow University, Suzhou, China; ^2^ Department of Ultrasound Medicine, The Second Affiliated Hospital of Soochow University, Suzhou, China; ^3^ Department of Pharmacy, The 960th Hospital of PLA, Jinan, China

**Keywords:** *Candida albicans*, tolerance, imidazole, aneuploidy, Hsp90, calcineurin, efflux

## Abstract

Antifungal resistance and antifungal tolerance are two distinct terms that describe different cellular responses to drugs. Antifungal resistance describes the ability of a fungus to grow above the minimal inhibitory concentration (MIC) of a drug. Antifungal tolerance describes the ability of drug susceptible strains to grow slowly at inhibitory drug concentrations. Recent studies indicate antifungal resistance and tolerance have distinct evolutionary trajectories. Superficial candidiasis bothers millions of people yearly. Miconazole has been used for topical treatment of yeast infections for over 40 years. Yet, fungal resistance to miconazole remains relatively low. Here we found different clinical isolates of *Candida albicans* had different profile of tolerance to miconazole, and the tolerance was modulated by physiological factors including temperature and medium composition. Exposure of non-tolerant strains with different genetic backgrounds to miconazole mainly induced development of tolerance, not resistance, and the tolerance was mainly due to whole chromosomal or segmental amplification of chromosome R. The efflux gene *CDR1* was required for maintenance of tolerance in wild type strains but not required for gain of aneuploidy-mediated tolerance. Heat shock protein Hsp90 and calcineurin were essential for maintenance as well as gain of tolerance. Our study indicates development of aneuploidy-mediated tolerance, not resistance, is the predominant mechanism of rapid adaptation to miconazole in *C. albicans*, and the clinical relevance of tolerance deserves further investigations.

## Introduction

*Candida albicans* is a common commensal fungus colonizing the human oropharyngeal cavity, gastrointestinal and vaginal tract and skins. It is part of the normal flora of the microbiota in approximately 50% of individuals (Nobile and Johnson, 2015). However, *C. albicans* is also a major fungal pathogen causing infections ranging from superficial skin and mucosal lesions to life-threatening infections with severe mortality rate of 40–60% (Pfaller and Diekema, 2007). The most common type of candidiasis is superficial infections of the mouth, vagina and skin. Imidazoles, such miconazole and clotrimazole, are widely used for topical treatments of fungal infections (Dias et al., 2013).

Miconazole (MCZ) is a synthetic imidazole derivative that has potent antifungal efficacy when being used topically against a wide variety of yeasts and dermatophytes (Sawyer et al., 1975; Ivanov et al., 2022). MCZ has two modes of action: the fungistatic mode through inhibition of 14-α-sterol demethylase, and the fungicidal mode through increasing intracellular reactive oxygen species, at least in part through inhibition of fungal catalase and peroxidase (Kobayashi et al., 2002; Thevissen et al., 2007). MCZ has been widely and successfully used for many candidal infections for more than 40 years (Regidor et al., 2023). The use of MCZ in medicine is primarily in topical forms. Common indications include vaginal and oral candidiasis, skin and nail infections due to *Trichophyton*, *Epidermophyton*, and *Pityrosporon* species, dermatomycosis, and onychomycosis (Regidor et al., 2023). The primary resistance mechanisms in *C. albicans* are an accumulation of mutations in *ERG11* or elevated copy number of *ERG11*, the gene encoding for the 14-α-sterol demethylase, as well as increased efflux from the cytoplasm by ATP-binding cassette (ABC) and major facilitator superfamily transporters (Fisher et al., 2022). Despite the wide availability and use of MCZ, fungal resistance to this drug remains relatively low (Barasch and Griffin, 2008).

In addition to resistance, other terms are also used to describe cellular responses to antifungal drugs, including tolerance, persistence and heteroresistance [reviewed in (Chen et al., 2023)]. Antifungal tolerance is defined as the “ability of a subpopulation of cells from a susceptible isolate to grow, albeit slowly, in the presence of drug concentrations above established minimum inhibitory concentrations (MICs)” (Berman and Krysan, 2020). Antifungal tolerance is a response distinct from antifungal resistance. Mechanistically, antifungal resistance is usually due to genetic/genomic mutations that directly affect the drug-target interactions. Antifungal tolerance depends upon diverse stress pathway responses, including heat shock responses, responses to amino acid starvation, kinases such as protein kinase C, and epigenetic processes (Rosenberg et al., 2018; Berman and Krysan, 2020). Recent studies from diverse research groups have consistently shown that the evolution of resistance and tolerance to azole drug fluconazole is dose-dependent, with low doses promoting the development of resistance and high doses leading to the emergence of tolerance (Todd et al., 2023; Yang et al., 2023). In addition to driving resistance, low-dose fluconazole also triggers the development of tolerance, although the frequency of mutations was much lower compared to high-dose treatment (Sun et al., 2023).

Disk diffusion assay (DDA) has been the mainstay for antimicrobial susceptibility testing in most institutions and hospitals. It is a relatively inexpensive, easy to use and flexible agar-based method which provides qualitative result for rapidly growing microorganisms. A microorganism is judged sensitive or resistant according to the diameter of the zone of inhibition (ZOI) of cultural growth, which correlates inversely with the MICs from standard dilution tests. However, growth inside the ZOI is ignored (Institute, C.a.L.S., 2009). Recent studies indicate DDA is an ideal method for measuring antifungal tolerance. Due to the nature of slow growth in the presence of supra-MIC concentrations of drugs, tolerance is usually evidenced by lawn growth inside ZOI after 24h incubation of the agar plates (Rosenberg et al., 2018; Xu et al., 2021; Sun et al., 2023; Yang et al., 2023). In order to quantify tolerance, a custom R script called *diskImageR* was created (Gerstein et al., 2016). The degree of drug resistance is determined by the radius of inhibition (RAD), and tolerance is determined by the fraction of growth (FoG) within the ZOI. Usually, 20% drug inhibition (RAD_20_ and FoG_20_) is used to measure resistance and tolerance, respectively (Gerstein et al., 2016; Rosenberg et al., 2018).

In *C. albicans*, tolerance to fluconazole has been well characterized (Rosenberg et al., 2018; Todd et al., 2023; Yang et al., 2023). Tolerance to other azoles, including posaconazole and ketoconazole, is also reported (Xu et al., 2021; Kukurudz et al., 2022). However, tolerance to MCZ is unexplored. In this study, we found MCZ tolerance in clinical isolates of *C. albicans* was dependent on strain background and was modulated by physiological factors including temperature and medium composition. Upon exposure to MCZ, non-tolerant cells mainly developed tolerance, not resistance, to MCZ. Interestingly, different selection force resulted in similar level of tolerance due to formation of aneuploids with same karyotype. Although the deletion of the efflux gene *CDR1* resulted in loss of tolerance in SC5314, the deletion strain could still develop tolerance. However, pharmacological inhibition of Hsp90 or genetic deletion of genes encoding subunits of calcineurin resulted in loss of tolerance as well as failure of development of tolerance, indicating both Hsp90 and calcineurin were essential for tolerance. This study indicates development of aneuploidy-mediated tolerance is the major response to MCZ in *C. albicans*, and the tolerance is dependent on *CDR1*-mediated efflux, Hsp90 and calcineurin.

## Materials and methods

### Strains and growth conditions

Strains used in this study are listed in **Supplementary Table 1**. Stock cultures were preserved in 25% glycerol and maintained at -80˚C. Cells were routinely grown in YPD media (1% [w/v] yeast extract, 2% [w/v] peptone and 2% [w/v] D-glucose) at 30˚C in a shaking incubator at 150–200 rpm. SDC agar plates contained 0.67% [wt/vol] yeast nitrogen base without amino acids, 2% [wt/vol] d-glucose, 0.2% [wt/vol] complete amino acid mixture, and 2% [wt/vol] agar. For solid medium, 2% [w/v] agar was added. For the selection of gene knockout strains, YPD agar containing 400 μg/mL nourseothricin (Werner BioAgents) medium was used (YPD+NAT). To evict the disruption cassette, yeast nitrogen base (YNB)–bovine serum albumin (BSA) (0.17% [w/v] YNB, 2% [w/v] D-glucose, 0.02% [w/v] BSA, 2% [w/v] agar) plates were used. Drugs were dissolved in dimethyl sulfoxide (DMSO) and stored at -20˚C.

### Construction of gene deletion strains

NAT1 flipper gene deletion cassette was amplified from plasmid pJK863 (Shen et al., 2005). Approximately 500 bp upstream and 500 bp downstream regions of the gene to be deleted was amplified from the genomic DNA of SC5314. The upstream region of each gene was then fused by PCR to the 5′ region of the cassette and the downstream region of the gene was fused by PCR to the 3′ region of the cassette, such that the two PCR products had an overlap of 500–1,000 bp within the cassette. All primer sequences used for plasmid and strain construction are listed in **Supplementary Table 2**. The upstream and downstream fusion products for each gene were then simultaneously transformed in *C. albicans* following the lithium acetate method (Wilson et al., 2000). Transformants were selected on YPD+NAT agar plates. The replacement of the gene with the NAT1 flipper cassette was confirmed by diagnostic PCR, using primers that annealed outside the flanking homology regions. The NAT1 flipper was then evicted by streaking the clones on YNB-BSA plates, which induces the Flp recombinase.

### Disk diffusion assay

The CLSI M44-A2 guidelines (Institute, C.a.L.S., 2009) for antifungal disk diffusion susceptibility testing were followed, with slight modifications. Strains were grown on YPD-agar plates, cell density was adjusted to 1 × 10^6^ cells/mL, and 100 μL of cell suspension was plated on plates. One paper disk (GE Healthcare, USA) supplemented with 50 μg miconazole was placed in the center of each plate. The plates were then incubated for 48 h then photographed. Each strain was evaluated in duplicate, with two independent biological replicates.

### Spot assay

Cells were suspended in distilled water and adjusted to 1x10^6^ cells/mL. 3 µL of 10-fold serial dilutions were spotted on YPD with or without drugs. The plates were incubated at 30˚C and photographed after 48 h. The assay was repeated twice, with independent experiments conducted at distinct time points.

### Obtaining miconazole adaptors

Cells were suspended in distilled water and adjusted to 1x10^7^ cells/mL. 100 µL of cell suspension were spread on YPD plates supplemented with MCZ. The plates were incubated at 30˚C for 5 days. 9–30 adaptors were randomly chosen.

### DNA-seq

Total DNA extraction and genomic DNA library preparation were performed as described previously (Yang et al., 2021). The final libraries were sequenced by BGISEQ-500. Raw fastq files were uploaded to YMAP (version 1.0) (http://lovelace.cs.umn.edu/Ymap/) (Abbey et al., 2014). Read depth was plotted as a function of chromosome position using the Assembly 22 version of the SC5314 reference genome (http://www.candidagenome.org/download/sequence/C_albicans_SC5314/Assembly22/current/C_albicans_SC5314_A22_current_chromosomes.fasta.gz).

## Results

### Characterization of miconazole tolerance in wild type strains

Two clinical isolates and one reference strain were tested for MCZ tolerance. SC5314 is the commonly used reference strain. YJB-T1891 and YJB-T490 were clinical isolates collected from Israeli patients (Yang et al., 2023). Two temperature conditions were tested: 30°C and 37°C. Two type of media was utilized: YPD and SDC. YPD is a rich, non-defined media that supports rapid growth. SDC is a more defined and controlled media. Two assays were performed: DDA and spot assay.

**Figure 1:**
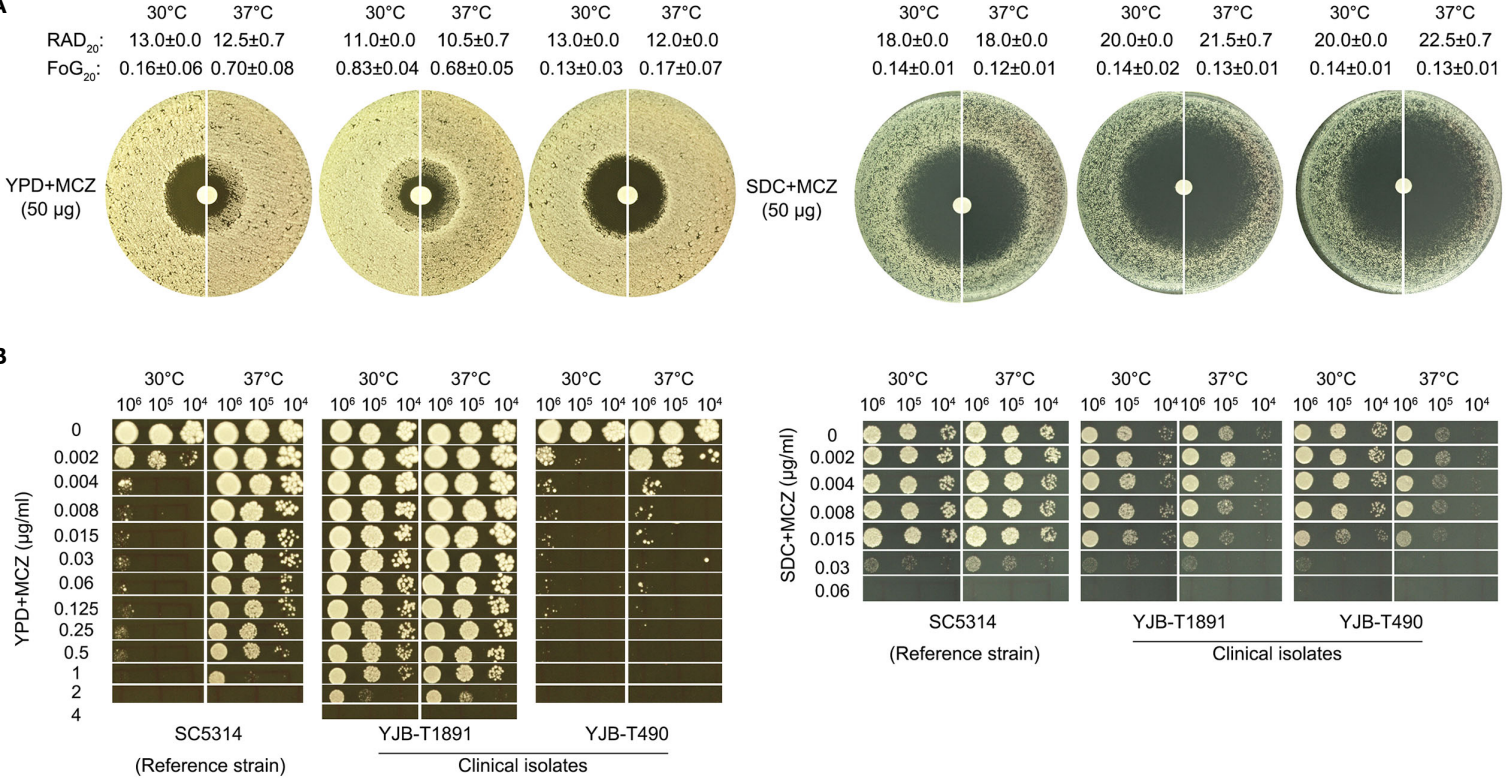
Miconazole tolerance in *C. albicans* clinical isolates is genetic background-dependent and is regulated by physiological factors. The clinical isolates YJB-T1891, SC5314, and YJB-T490 were tested with disk diffusion assays **(A)** and spot assays **(B)**. Both assays were performed at 30°C and 37°C on YPD-agar and SDC-agar. In disk diffusion assays, the disks contained 50 μg MCZ. In spot assays, the plates were supplemented with 0.002–4 μg/mL MCZ as indicated in the figure. The plates were incubated for 48h then photographed.

In DDAs performed on YPD-agar, at both temperatures, YJB-T1891 had colonies growing inside the ZOI and YJB-T490 had clear ZOI. SC5314 had clear ZOI at 30°C and colonies growing inside the ZOI at 37°C. In addition, SC5314 and YJB-T490 had smaller ZOI at 37°C than at 30°C (**Figure 1A** left panel). In DDAs performed on SDC-agar, all strains displayed clear ZOI. YJB-T1891 and YJB-T490 had bigger ZOI at 37°C than at 30°C (**Figure 1A** right panel).

Analysis of DDA plates with *diskimageR* indicated that, on YPD-agar, the RAD_20_ values of SC5314 at 30°C and 37°C were 13.0 ± 0.0 and 12.5 ± 0.7, respectively. The RAD_20_ values of YJB-T1891 at 30°C and 37°C were 11.0 ± 0.0 and 10.5 ± 0.7, respectively. And the RAD_20_ values of YJB-T490 at 30°C and 37°C were 13.0 ± 0.0 and 12.0 ± 0.0, respectively. Thus, when tested with YPD-agar, temperature condition did not alter extent of resistance. On SDC-agar, the RAD_20_ values of SC5314 at both 30°C and 37°C were 18.0 ± 0.0. The RAD_20_ values of YJB-T1891 at 30°C and 37°C were 20.0 ± 0.0 and 21.5 ± 0.7, respectively. And the RAD_20_ values of YJB-T490 at 30°C and 37°C were 20.0 ± 0.0 and 22.5 ± 0.7, respectively (**Figure 1A**). Thus, when tested with SDC-agar, strains had slightly increased resistance at lower temperature.

As compared to the slight change of RAD, the change of FoG was more obvious on YPD-agar. The FoG_20_ values of SC5314 at 30°C and 37°C were 0.16 ± 0.06 and 0.70 ± 0.08, respectively. The FoG_20_ values of YJB-T1891 at 30°C and 37°C were 0.83 ± 0.04 and 0.68 ± 0.05, respectively. The FoG_20_ values of YJB-T1891 at 30°C and 37°C were 0.13 ± 0.03 and 0.17 ± 0.07, respectively (**Figure 1A**).

In spot assays performed on YPD-agar, strains were tested for the ability of growing in the presence of a wide range of MCZ concentrations (0.002–4 μg/mL). At both temperatures, YJB-T1891 could tolerate 1 μg/mL MCZ. SC5314 could tolerate 1 μg/mL MCZ only at 37°C, while it was obviously inhibited by 0.002 μg/mL MCZ at 30°C. YJB-T490 could only tolerate 0.002 and 0.004 μg/mL MCZ at 30°C and 37°C, respectively (**Figure 1B** left panel). On SDC-agar plates, at both temperatures, all strains could only tolerate 0.015 μg/mL MCZ. Both YJB-T1891 and YJB-T490 grew better at 30°C than at 37°C on plates supplemented with or without MCZ (**Figure 1B** right panel).

Thus, MCZ tolerance was strain background-dependent and was modulated by physiological factors including temperature and medium composition.

### Exposure of SC5314 to miconazole induces gain of tolerance, not resistance, to miconazole

**Figure 2:**
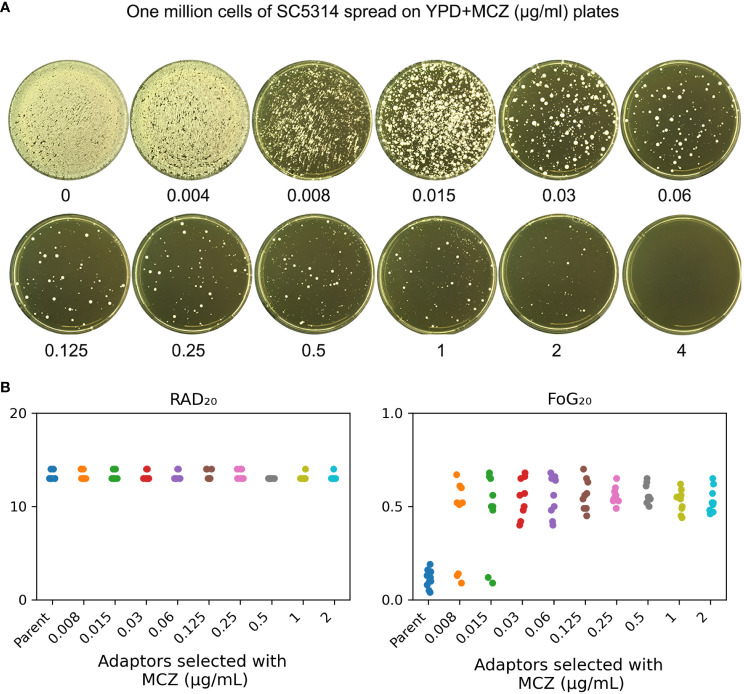
Exposure to high amount of miconazole induces tolerance. **(A)** Approximately one million cells of SC5314 were spread on YPD-agar plates supplemented with miconazole. Drug concentrations were shown in the figure. The plates were incubated at 30°C for 5 days then photographed. **(B)** Randomly nine adaptors were chosen from each drug plate and tested with disk diffusion assay using disks containing 50 μg MCZ. RAD_20_ and FoG_20_ values were calculated using *diskImageR* script. Sources of the adaptors are indicated in the figure. For the parent, 10 individual colonies were tested as 10 biological replicates.

In order to investigate how non-tolerant isolate adapts to miconazole, SC5314 was exposed to high concentrations of MCZ (0.004–4 μg/mL) and the plates were incubated at 30°C (**Figure 2A**). After 5 days, randomly 9 colonies were chosen from each of plates supplemented with 0.008–2 μg/mL MCZ. All the 81 adaptors were tested for MCZ tolerance by DDAs (**Supplementary Figure 1**). Only 3 adaptors derived from 0.008 μg/mL MCZ (#3, #7 and #9), and another 2 adaptors derived from 0.015 μg/mL MCZ (#4 and #5) were not tolerant. All the other 76 adaptors were tolerant. Of note, none of the 81 adaptors displayed altered radius of the inhibition zone, indicating they were not resistant.

The *diskImageR* analysis indicates that the parent had RAD_20_ and FoG_20_ values of 13.2 ± 0.42 and 0.11 ± 0.05, respectively, while the adaptors had RAD_20_ and FoG_20_ values of 13.2 ± 0.41 and 0.53 ± 0.11, respectively (**Figure 2B**). The results of the Student’s t-test show that there was no significant difference in RAD_20_ between the parent and the adaptors (p>0.05), whereas the adaptors had significantly higher FoG_20_ values than the parent (p<0.001).

**Figure 3:**
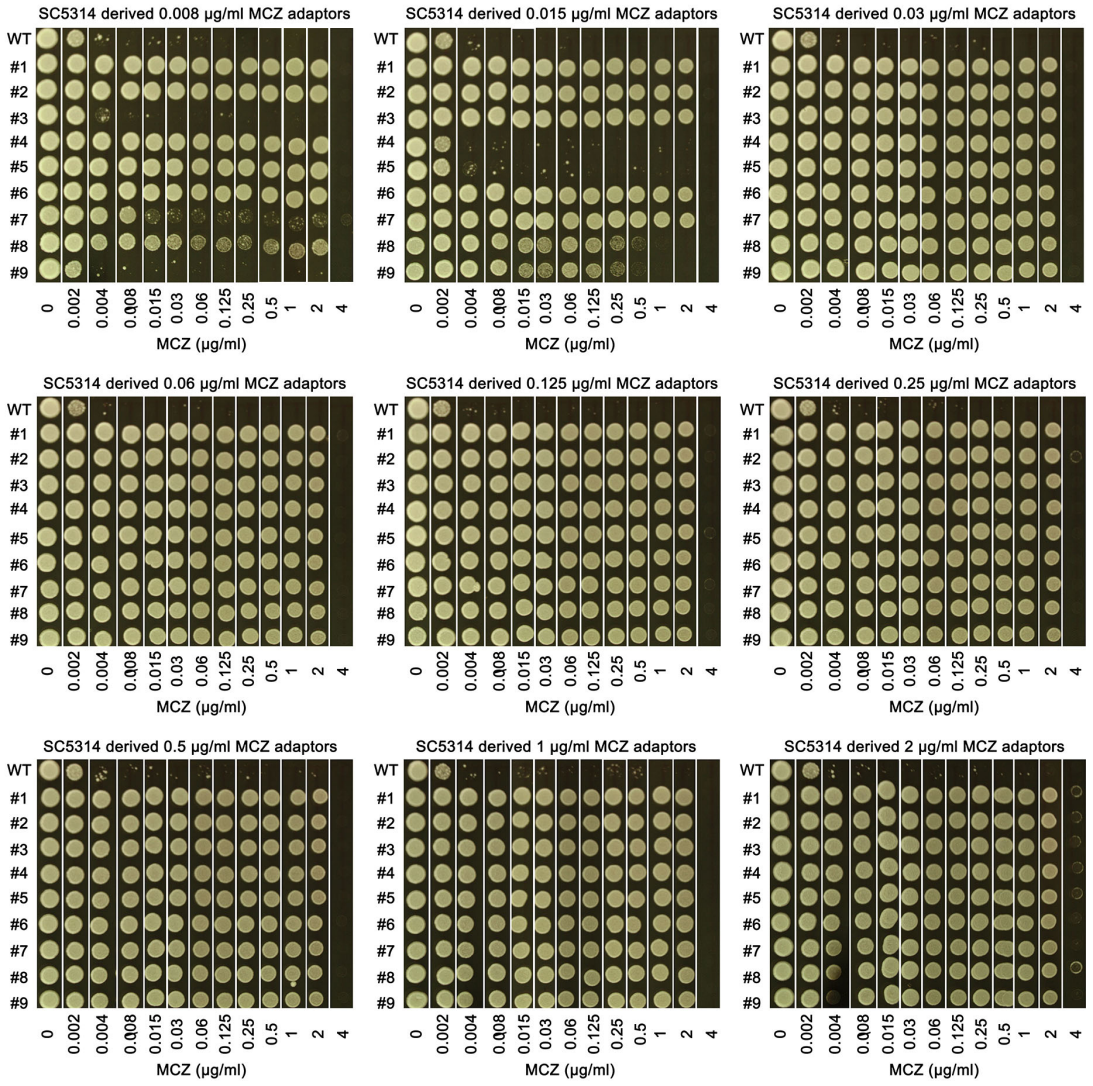
Assessing extent of miconazole tolerance in SC5314 derived adaptors. 81 adaptors derived from SC5314 were tested with spot assay. The sources of the 81 adaptors were indicated in the figure. WT indicates SC5314. YPD-agar plates supplemented with 0.002–4 μg/mL miconazole as indicated in the figure. 3 μL of cells at a density of 1x10^6^ cells/mL were spotted on the plates. The plates were incubated at the designated temperatures for 48 h then photographed.

We asked to what extent the adaptors could tolerate MCZ. Spot assay was performed on YPD-agar plates supplemented with 0.002–4 μg/mL MCZ. Growth of the parent SC5314 and 4 non-tolerant adaptors, #3 and #9 derived from 0.008 μg/mL MCZ, as well as #4 and #5 derived from 0.015 μg/mL MCZ, could only grow in the presence of 0.002 μg/mL MCZ. Another non-tolerant adaptor, #7 derived from 0.008 μg/mL MCZ, could only grow in the presence of 0.008 μg/mL MCZ. Two tolerant adaptors, #8 and #9 derived from 0.015 μg/mL MCZ plate, could grow in the presence of 0.25 μg/mL MCZ, and all the other 74 tolerant adaptors could grow on plates containing 2 μg/mL MCZ, but none of them could grow in the presence of 4 μg/mL MCZ (**Figure 3**). The difference in the levels of tolerance might be due to different genetic or genomic changes happened in different adaptors.

### Tolerance is due to formation of chromosome R trisomy

**Figure 4:**
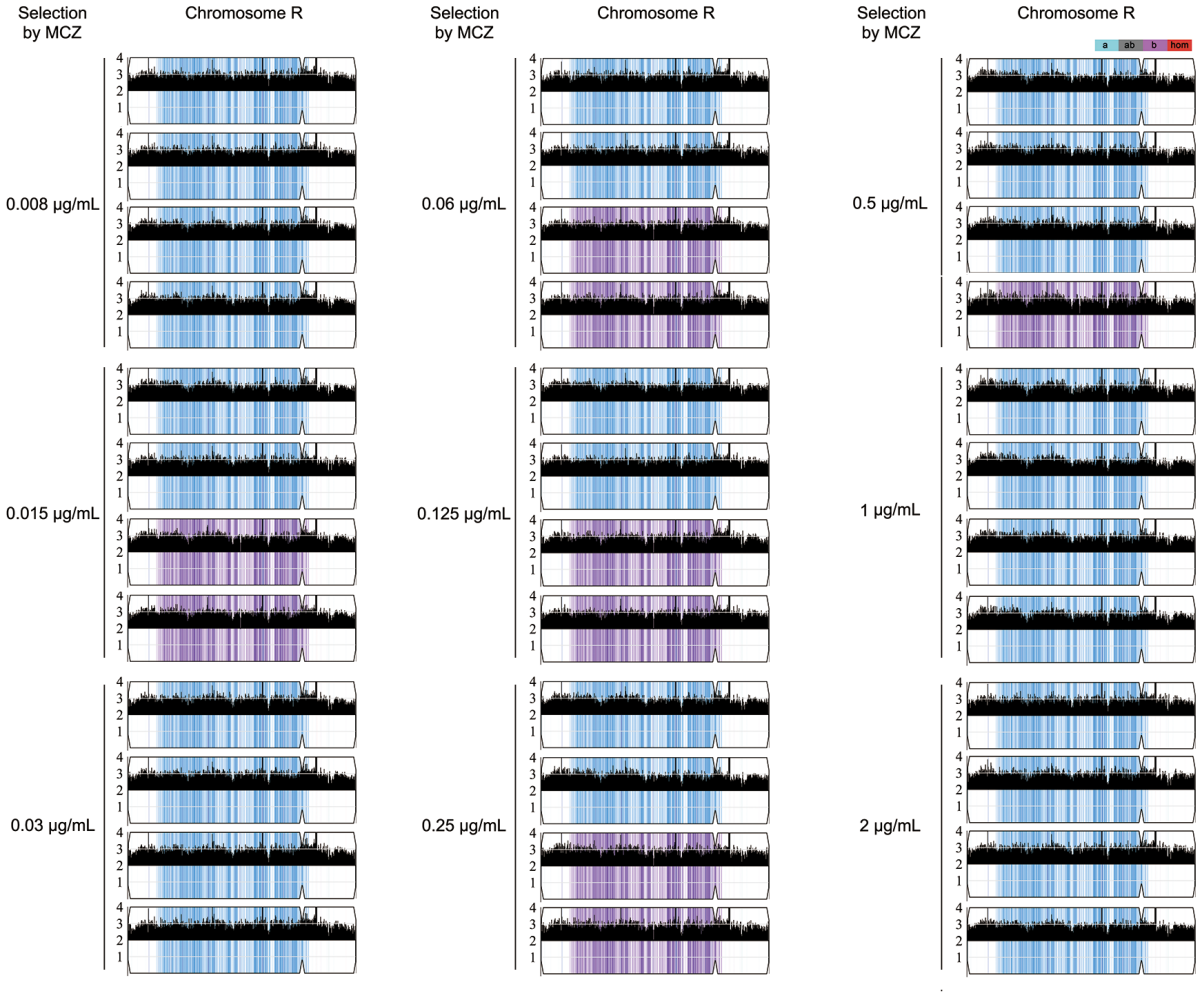
Karyotypes of miconazole tolerant adaptors. Randomly 36 adaptors derived from exposure to 0.008–2 μg/mL miconazole were sequenced. The sequencing data was visualized using Ymap. Read depth (normalized to that of the diploid parent) is shown on the y-axis on a log_2_ scale converted to absolute copy numbers (1–4). Allelic ratios (A:B) are color-coded: gray, 1:1 (A/B); cyan, 1:0 (A or A/A); magenta, 0:1 (B or B/B); purple, 1:2 (A/B/B); blue, 2:1 (A/A/B).

Randomly 4 tolerant adaptors which were derived from each concentration of MCZ (0.008–2 μg/mL) and could tolerate 2 μg/mL MCZ, were sequenced. All the 36 sequenced adaptors had chromosome R trisomy (ChrRx3): 27, and 9 adaptors had duplication of A and B holomog, respectively (**Figure 4**; **Supplementary Figure 2**). Thus, under different selection force, the same aneuploidy appeared and thereby conferring similar extent of MCZ tolerance.

Two less tolerant adaptors (#8 and #9) derived from 0.015 μg/mL MCZ plate were also sequenced. Both were euploid (**Supplementary Figure 3**).

### *CDR1* is required for maintenance of miconazole tolerance

Increased efflux is one of the major mechanisms of drug resistance across the kingdoms. But essentiality of efflux in maintenance of tolerance is largely unexplored. In *C. albicans* genome, *CDR1* and *CDR2* encode multidrug transporter of the ATP-binding cassette (ABC) superfamily, and *MDR1* encodes multidrug resistance protein of the major facilitator superfamily (MFS). *TAC1* encodes a Zn(2)-Cys(6) binuclear cluster type transcriptional activator of drug-responsive genes including the ABC drug transporters, *CDR1* and *CDR2*. In this study, both alleles of *CDR1*, *CDR2*, *MDR1* and *TAC1* were deleted from SC5314. The deletion strains as well as the wild type were tested with DDAs and spot assays.

**Figure 5:**
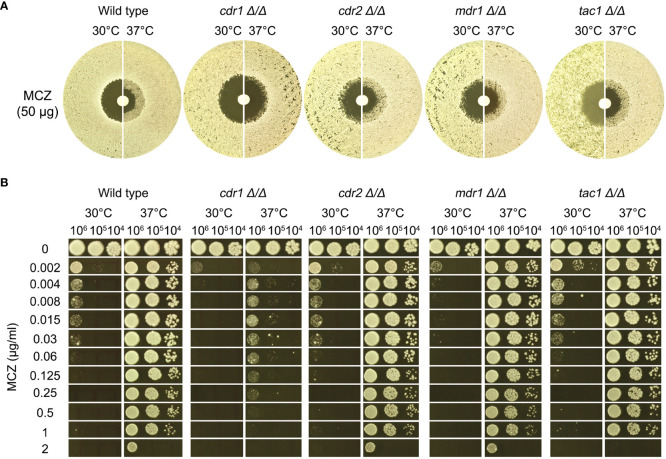
Role of efflux genes in miconazole tolerance. Efflux genes *CDR1*, *CDR2*, and *MDR1* were deleted from SC5314. The strains were tested for miconazole tolerance with disk diffusion assays **(A)** and spot assays **(B)**. YPD-agar medium was used, and the assays were performed at 30°C and 37°C as indicated in the figure. In **(B)** 3 μL of cells at densities of 10^6^, 10^5^ and 10^4^ cells/ml were spotted on the plates. In both assays, the plates were incubated at the designated temperatures for 48 h then photographed.

In DDAs, the wild type, *cdr2* Δ/Δ, *mdr1* Δ/Δ and *tac1* Δ/Δ strains had clear ZOI at 30°C and had colonies growing inside the ZOI at 37°C, while *cdr1* Δ/Δ strain also had clear ZOI at 30°C and had obviously less colonies growing inside the ZOI at 37°C (**Figure 5A**).

In spot assays, the wild type and the strains with deletions of *CDR2*, *MDR1* and *TAC1* could tolerate 1 μg/mL MCZ at 37°C and the growths were obviously inhibited by 0.002 μg/mL MCZ at 30°C. Growth of *cdr1 Δ/Δ* strain was inhibited by 0.002 μg/mL MCZ at both 30°C and 37°C (**Figure 5B**).

Thus, *CDR1*, but not *CDR2*, *MDR1* or *TAC1*, was required for maintaining MCZ tolerance at 37°C.

### *CDR1* is not required for development of miconazole tolerance

**Figure 6:**
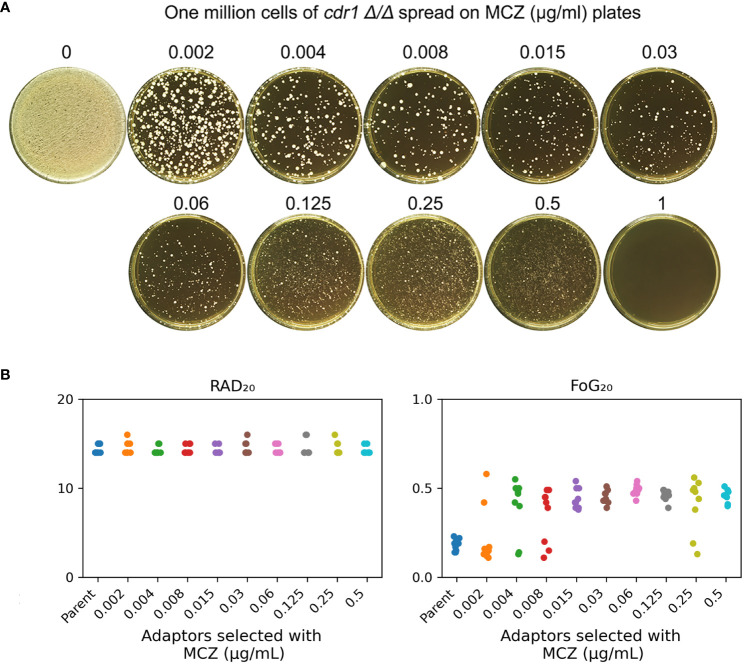
*cdr1 Δ/Δ* strain adapts to miconazole via gain of tolerance. **(A)**, approximately one million cells of SC5314 derived *CDR1* deletion strain were spread on YPD-agar plates supplemented with miconazole. The drug concentrations were indicated in the figure. The plates were incubated at 30°C for 5 days then photographed. **(B)**, 81 adaptors were tested with disk diffusion assays. The disks contained 50 μg miconazole. RAD_20_ and FoG_20_ values were calculated using *diskImageR* script. Sources of the adaptors are indicated in the figure. For the parent, 10 individual colonies were tested as 10 biological replicates.

We asked if *CDR1* was essential for gain of miconazole tolerance. The *cdr1 Δ/Δ* strain was spread on YPD plates supplemented with MCZ. On plates containing 0.002 – 0.5 μg/mL MCZ, colonies appeared within 5 days (**Figure 6A**). Randomly 9 adaptors from each drug plate were tested with DDAs. Among the 81 adaptors, only 7, 2, 3 and 2 adaptors derived from 0.002, 0.004, 0.008 and 0.25 μg/mL MCZ, respectively, were not tolerant to MCZ. Of note, like SC5314 derived adaptors, none of the 81 adaptors derived from *cdr1 Δ/Δ* strain had altered size of radius of inhibition zone (**Supplementary Figure 4**). The *diskImageR* analysis indicates that the parent *cdr1 Δ/Δ* strain had RAD_20_ and FoG_20_ values of 14.4 ± 0.52 and 0.18 ± 0.03, respectively, while the adaptors had RAD_20_ and FoG_20_ values of 14.6 ± 0.60 and 0.41 ± 0.13, respectively (**Figure 6B**). The results of the Student’s t-test show that there was no significant difference in RAD_20_ between the parent and the adaptors (p>0.05), whereas the adaptors had significantly higher FoG_20_ values than the parent (p<0.001). Thus, exposure of *cdr1 Δ/Δ* strain to MCZ mainly induced development of tolerance.

**Figure 7:**
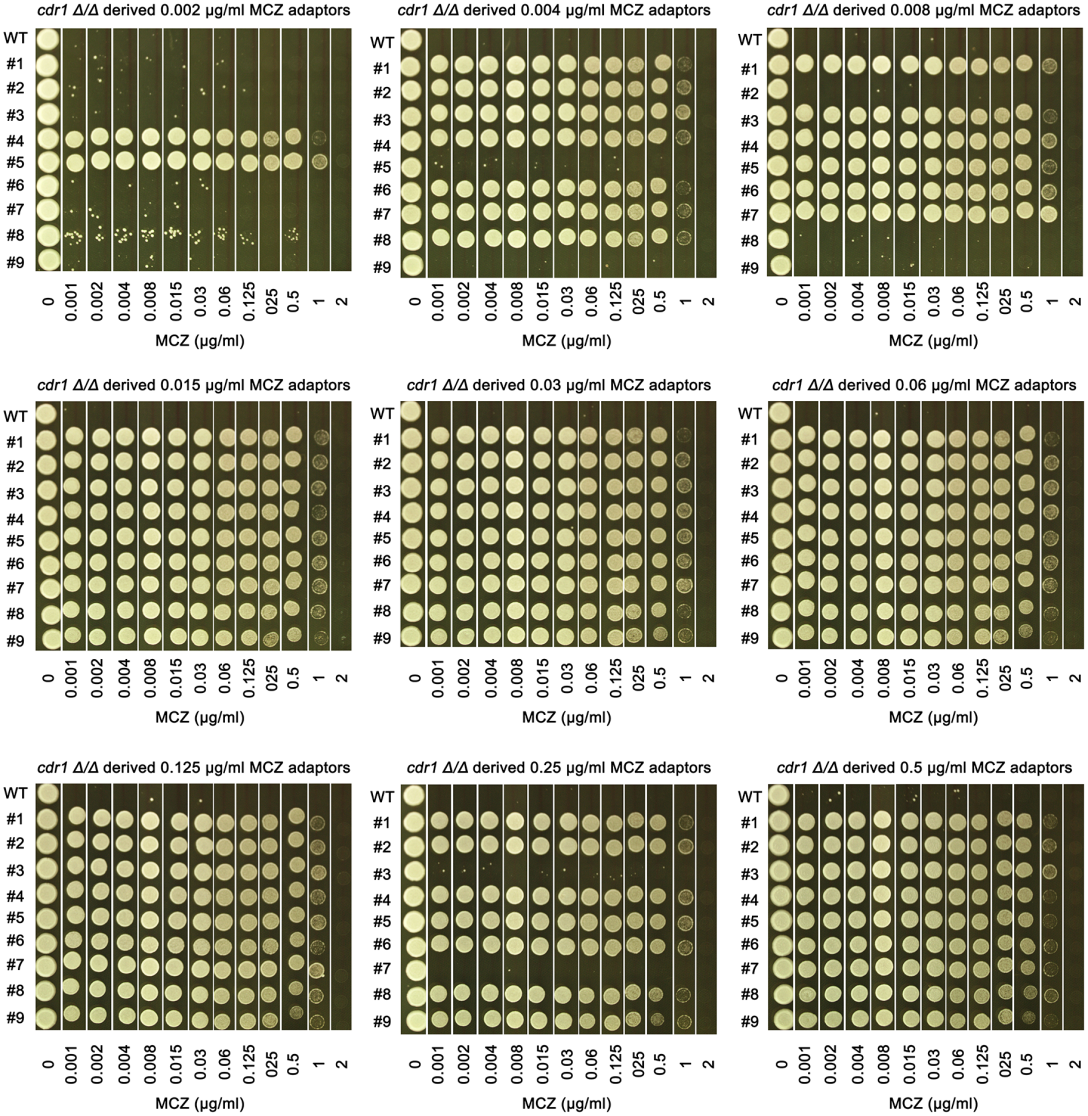
Assessing extent of miconazole tolerance in *cdr1 Δ/Δ* derived adaptors. Spot assay was performed on YPD-agar plates supplemented with wide range of miconazole concentrations as indicated in the figure. The sources of the adaptors were indicated in the figure. WT indicates *cdr1 Δ/Δ* strain. The plates were incubated at 30°C for 2 days then photographed.

We asked to what extent the *cdr1 Δ/Δ* derived adaptors could tolerate MCZ. The 14 non-tolerant adaptors as well as the wild type were similarly inhibited by 0.001 μg/mL MCZ, while most of the 67 tolerant adaptors could grow in the presence of 1 μg/mL MCZ. There was no obvious difference in the extent of tolerance among the tolerant adaptors (**Figure 7**). We sequenced 2 tolerant adaptors (#4 and #5) derived from 0.002 μg/mL MCZ and 4 tolerant adaptors (#1-#4) derived from 0.5 μg/mL MCZ. All 6 adaptors had trisomy of ChrR. Notably, there was no evidence of biased amplification of either ChrR homolog. Three adaptors exhibited amplification of the A homolog (AAB), while the remaining three adaptors showed amplification of the B homolog (ABB) (**Supplementary Figure 3**).

### Hsp90 is required for miconazole tolerance and resistance

Heat shock protein 90 (Hsp90) is a highly conserved vital chaperone protein required for the stability and/or folding of hundreds of client proteins. In yeasts, Hsp90 potentiates development of antifungal resistance (Cowen et al., 2006) and is required for the maintenance of tolerance to azoles (Rosenberg et al., 2018; Xu et al., 2021). In order to investigate essentiality of Hsp90 for MCZ tolerance, DDAs were performed using YPD-agar plates supplemented with and without NVP-HSP990. NVP-HSP990 is a potent and selective synthetic small-molecule Hsp90 inhibitor (Menezes et al., 2012).

**Figure 8:**
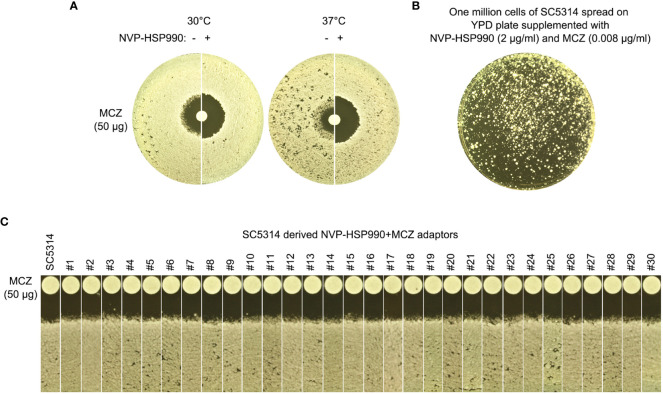
Essentiality of Hsp90 for development of miconazole resistance and tolerance. **(A)** Disk diffusion assays were performed using YPD-agar plates with (+) or without **(-)** 2 μg/mL NVP-HSP990. The disks contained 50 μg miconazole. The test strain was SC5314. The plate was incubated at 30°C for 2 days then photographed. **(B)** Approximately one million cells of SC5314 were spread on YPD-agar plate supplemented with 2 μg/mL NVP-HSP990 and 0.008 μg/mL miconazole. The plate was incubated at 30°C for 5 days then photographed. **(C)** Randomly 30 adaptors (#1-#30) were tested with disk diffusion assays. The disks contained 50 μg miconazole. The plates were incubated at 30°C for 2 days then photographed.

At both 30°C and 37°C, addition of NVP-HSP990 resulted in bigger ZOI. At 37°C, addition of NVP-HSP990 resulted in disappearance of growth inside the ZOI (**Figure 8A**). Thus, inhibition of Hsp990 caused decreased tolerance and resistance to MCZ.

We asked if cells could still develop resistance or tolerance when Hsp90 was inhibited. Approximately one million cells of SC5314 were spread on YPD-agar plate supplemented with NVP-HSP990 and MCZ (**Figure 8B**). 5 days later, randomly 30 adaptors were chosen. DDAs indicated all the adaptors had clear ZOI, and all adaptors had similar size of ZOI as compared to the parent SC5314 (**Figure 8C**). Thus, cells with suppressed Hsp90 could not develop resistance or tolerance to MCZ.

### Calcineurin is required for miconazole tolerance and resistance

Calcineurin is a conserved Ca^2+^-calmodulin activated protein phosphatase and is involved in calcium- dependent signaling and regulation of several important cellular processes including evolution of drug resistance in both yeasts and filamentous fungi (Juvvadi et al., 2017). Calcineurin is a heterodimer comprised of a catalytic subunit and a regulatory subunit. In *C. albicans* genome, these two subunits are encoded by *CMP1* and *CNB1*, respectively. *CRZ1* encodes a C2H2 zinc-finger domain transcription factor which is the downstream target of calcineurin (Onyewu et al., 2004). Both alleles of these three genes were deleted from SC5314 and the deletion strains were evaluated for MCZ tolerance.

**Figure 9:**
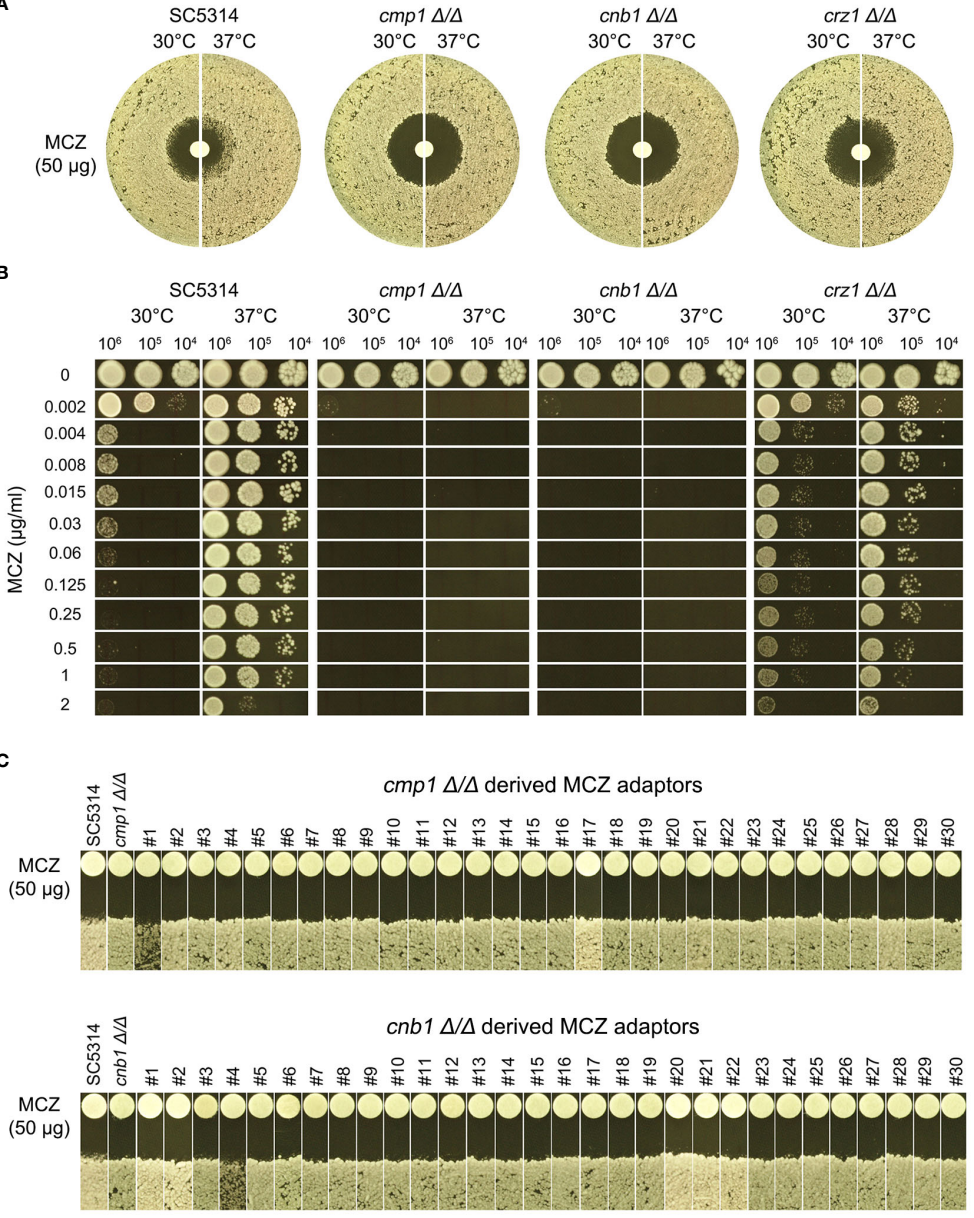
Essentiality of calcineurin for development of miconazole resistance and tolerance. *CMP1*, *CNB1* and *CRZ1* were deleted from SC5314. Tolerance to miconazole was evaluated by disk diffusion assays **(A)** and spot assay **(B)**. They assays were performed at 30°C and 37°C as indicated in the figure. In **(C)** *cmp1 Δ/Δ* and *cnb1 Δ/Δ* strains were spread on YPD-agar plates supplemented with 0.004 μg/mL miconazole. Randomly 30 adaptors were chosen and tested with disk diffusion assays.

In DDAs, *cmp1 Δ/Δ* and *cnb1 Δ/Δ* strains had clear ZOI on YPD at 37°C, while *crz1 Δ/Δ* strain still had colonies growing inside the ZOI, albeit less obvious than wild type (**Figure 9A**, top panel). In spot assays, growth of *cmp1 Δ/Δ* and *cnb1 Δ/Δ* strains was completely inhibited by 0.002 μg/mL MCZ at both 30°C and 37°C while *crz1 Δ/Δ* strain could tolerate 1 μg/mL MCZ at 37°C. Surprisingly, *crz1 Δ/Δ* strain was more tolerant than wild type at 30°C (**Figure 9B** bottom panel). Thus, calcineurin was required for maintaining MCZ tolerance at 37°C, but Crz1 was not required.

We asked if *cmp1 Δ/Δ* and *cnb1 Δ/Δ* strains could develop MCZ tolerance or resistance. Approximately one million cells of the deletion strains were spread on YPD-agar plates supplemented with 0.004 μg/mL MCZ. 5 days later, randomly 30 colonies were chosen. DDAs indicated all adaptors derived from *cmp1 Δ/Δ* and *cnb1 Δ/Δ* had clear ZOI, and none had obvious altered size of ZOI as compared to the parent (**Figure 9C**). Thus, strains with deletion of *CMP1* and *CNB1* failed to develop MCZ tolerance or resistance.

### Exposure of non-tolerant clinical isolate to miconazole induces gain of tolerance mediated by segmental or whole chromosomal amplification of chromosome R

**Figure 10:**
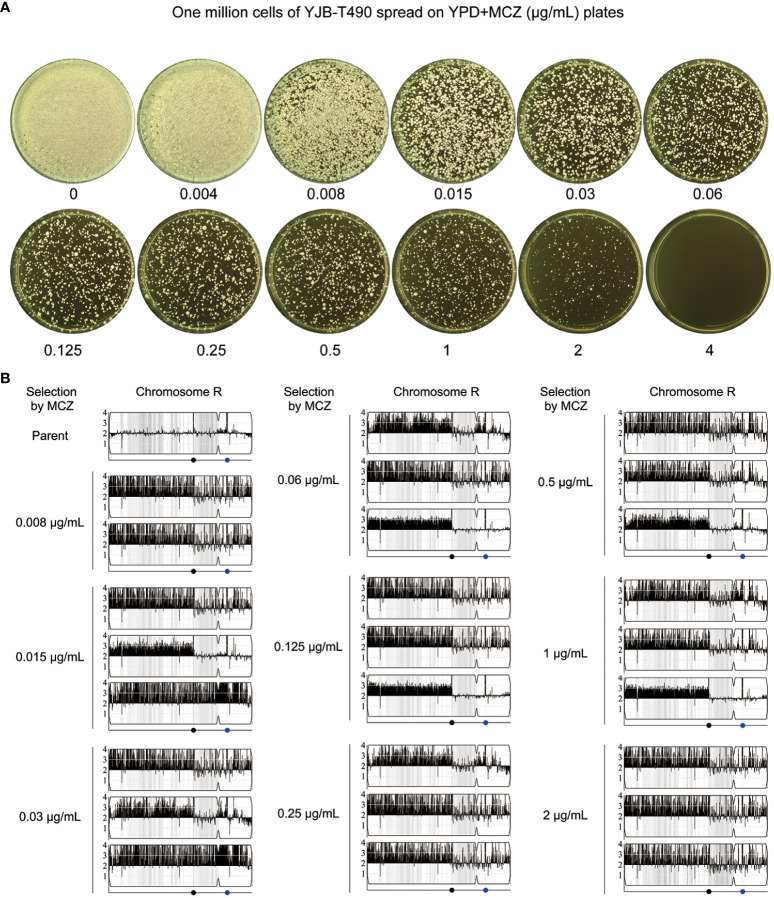
Segmental and whole chromosome amplification of chromosome R drives rapid adaptation of a clinical isolate to miconazole. **(A)** Approximately one million cells of YJB-T490 were spread on YPD-agar plates supplemented with various concentrations of MCZ. The plates were incubated at 30°C for 3 days then photographed. **(B)** Randomly selected MCZ-tolerant adaptors from each MCZ-containing plate were subjected to whole-genome sequencing. The sources and karyotypes of the sequenced adaptors are shown in the figure. Black dot indicates the locus of MRS (Major Repeat Sequence). The blue dot indicates the locus of rDNA.

We investigated whether the non-tolerant clinical isolate YJB-T490 adapted to MCZ in a similar manner to the reference strain SC5314. Approximately one million cells of YJB-T490 were spread on YPD-agar plates supplemented with 0.004–4 μg/mL MCZ (**Figure 10A**). From each of the plates with 0.008–2 μg/mL MCZ, randomly 9 adaptors were tested. In total, 81 adaptors were tested. Among them, 62 adaptors had obvious growth inside the ZOI, and 19 adaptors had clear ZOI. None of them had reduced ZOI (**Supplementary Figure 5A**). The *diskImageR* analysis indicates that the parent YJB-T490 strain had RAD_20_ and FoG_20_ values of 13.1 ± 0.31 and 0.18 ± 0.04, respectively, while the adaptors had RAD_20_ and FoG_20_ values of 13.3 ± 0.45 and 0.49 ± 0.19, respectively (**Supplementary Figure 5B**). The results of the Student’s t-test show that there was no significant difference in RAD_20_ between the parent and the adaptors (p>0.05), whereas the adaptors had significantly higher FoG_20_ values than the parent (p<0.001). Spot assay verified that all the 62 tolerant adaptors could grow in the presence of 2 μg/mL MCZ. The parent YJB-T490 and the 19 non-tolerant adaptors could only grow in the presence of 0.004 μg/mL MCZ (**Supplementary Figure 6**).

We randomly sequenced 26 tolerant adaptors. Two adaptors (#2 and #8) were derived from 0.008 μg/mL MCZ, and three adaptors were derived from each of the 0.015–2 μg/mL MCZ plates. All the 26 adaptors were aneuploid of ChrR. 24 adaptors had the same segmental trisomy of ChrR. The segment ranged from the left telomere to the MRS (Major Repeat Sequence) region. 2 adaptors had whole chromosomal trisomy of ChrR (**Figure 10B**; **Supplementary Figure 7**).

Thus, similar to the reference strain SC5314, the clinical isolate YJB-T490 also adapted to MCZ via gain of tolerance. However, although whole chromosomal trisomy of ChrR happened, the major genomic change was segmental amplification of ChrR.

## Discussion

In this study, we investigated the overlooked MCZ tolerance. We found intrinsic MCZ tolerance was strain-dependent and was modulated by physiological factors. We found acquiring ChrRx3-mediated tolerance was the predominant mechanism of rapid adaptation to MCZ in strains with different genetic backgrounds. We also demonstrated Hsp90 and calcineurin were essential for maintenance and acquisition of MCZ tolerance. *CDR1* was required for maintenance of tolerance in wild type strain but not required for ChrRx3-mediated evolution of tolerance.

Recent studies indicate evolution of resistance to fluconazole usually results in step-wise increase in MIC, while evolution of tolerance confers abrupt (from 2 μg/mL to higher than 128 μg/mL) increased ability of dose-independent growth in the presence of supra-MIC amounts of fluconazole, albeit the MIC remains unchanged (Yang et al., 2023). Temperature-modulated tolerance to ketoconazole also enables growth in the presence of approximately 2048 fold higher concentration of ketoconazole (from 0.015 μg/mL to 32 μg/mL) on agar plates (Xu et al., 2021). In this study, we also found, compared to lower temperature (30°C), higher temperature (37°C) enabled growth of SC5314 in the presence of 256-fold higher concentration of MCZ (from 0.004 μg/mL to 1 μg/mL) on YPD-agar plates, but the radius of the ZOI only displayed negligible change, indicating temperature mainly modulated tolerance, not resistance, to MCZ. Together, these findings support the hypothesis that tolerance is a physiological response that confers phenotypic leap.

We found medium modulated MCZ tolerance profile in clinical isolates, including YJB-T1891 and SC5314, which were classified as intrinsic tolerant and temperature enhanced tolerant to fluconazole on YPD-agar plates, respectively, in previous study (Yang et al., 2023). But on SDC-agar plates, both isolates were not tolerant. It remains unclear why medium composition regulates MCZ tolerance. It seems the medium effect is drug-specific, since previous studies indicate SC5314 is tolerant to ketoconazole but not tolerant to fluconazole on SDC-agar plates at both 30°C and 37°C (Xu et al., 2021; Yang et al., 2023). Growth medium also regulates drug resistance and biofilm formation in both bacteria and yeasts (Huys et al., 2002; Nayak et al., 2002; Ene et al., 2012; Serrano-Fujarte et al., 2015; Wijesinghe et al., 2019).

Antifungal tolerance and resistance appear with distinct evolutionary dynamics and trajectories. Low and high drug concentrations mainly drive development of resistance and tolerance, respectively. Resistance to azoles in clinical isolates is mainly due to amplification of the left arm of chromosome 5, while tolerance to azoles *in vitro* is mainly due to trisomy of chromosome R (Selmecki et al., 2006; Kukurudz et al., 2022; Todd et al., 2023; Yang et al., 2023). Consistently, here we found exposure of both wild type strain SC5314 and *cdr1 Δ/Δ* strain to high amount of MCZ mainly induced tolerance, and none of the adaptors displayed gain of resistance. Furthermore, we found development of MCZ tolerance in the reference strain SC5314 and one clinical isolate (YJB-T490) was mainly due to trisomy of whole ChrR or large proportion of ChrR. Thus, this study indicates trisomy of chromosome R mediates tolerance induced by imidazoles.

The mechanisms underlying the acquisition of MCZ tolerance have yet to be identified. Resistance to azoles often arises from alterations in the target enzyme and increased efflux of the drug from the fungal cell. These mechanisms are also applicable to antifungal tolerance. For example, fluconazole-tolerant isolates of *Candida albicans* often exhibit reduced intracellular accumulation of the drug. This reduction can be due to increased efflux or decreased uptake of the drug (Rosenberg et al., 2018). ChrR encodes several efflux genes (e.g.,*ADP1*, *MDL1*, *TPO4*), and *ERG* genes (e.g., *ERG25*, *ERG27*). It will be interesting to test whether overexpression of these genes alone or in combination phenocopies the effect of ChrR trisomy on tolerance.

The efflux gene *CDR1*, but not *CDR2* or *MDR1*, is partially required for maintenance of ketoconazole tolerance, but it is unclear if *CDR1* is required for development of ketoconazole tolerance (Xu et al., 2021). Here we found *CDR1* was also required for maintenance of temperature modulated MCZ tolerance. However, *CDR1* deletion strain could still develop tolerance upon exposure to MCZ, and the tolerance was mediated mainly by ChrRx3, indicating CDR1 was not essential for gain of aneuploidy-mediated tolerance. In addition, this study found *TAC1*, the gene encoding the master transcription factor regulating the expression of *CDR1* and *CDR2*, was not required for maintenance of MCZ tolerance. It is unclear whether there are other efflux genes required for maintenance and/or development of MCZ tolerance.

In addition to *Cdr1*, we found pharmacological inhibition of Hsp90 and genetic deletion of genes encoding subunits of calcineurin also caused loss of MCZ tolerance. Furthermore, cells with compromised function of Hsp90 or deletions of calcineurin subunit genes fail to develop tolerance when exposed to high concentrations of MCZ. Of note, the survived colonies were not resistant neither, indicating Hsp90 and calcineurin were essential for maintenance and gain of MCZ tolerance. In yeasts, aneuploidy often leads to proportional transcriptional and translational change of genes on aneuploid chromosomes, thereby eliciting proteotoxic stress and impairing cellular protein folding (Oromendia et al., 2012). Accumulation of unfolded proteins and inhibition of ergosterol synthesis in the endoplasmic reticulum stimulate an influx of Ca^2+^, which promotes cell survival by activating calcineurin (Bonilla et al., 2002). In the genome of *C. albicans*, ChrR is either the longest or the second longest chromosome, depending on the size of the rDNA. We propose that Hsp90 and calcineurin are essential for surviving proteotoxic stress in ChrR trisomy cells, and that their inhibition prevents the occurrence of ChrR trisomy.

It will be interesting to investigate whether Hsp90 and calcineurin are also essential for evolution of MCZ resistance. Previous study indicated that long-time exposure to sub-MIC concentrations of fluconazole mainly induced resistance (Yang et al., 2023). Thus, one approach can be doing daily passage of SC5314 in YPD broth supplemented with Hsp90 inhibitor NVP-HSP990, or calcineurin inhibitor cyclosporin A as well as low amount of MCZ.

While the antifungal potency of azoles may be compromised by the development of resistance and tolerance, it can be potentiated by certain adjuvants, including inhibitors of Hsp90, calcineurin, sphingolipid biosynthesis, and protein kinase C [reviewed in (Yang and Berman, 2024)]. A recent study by Ene et al. revealed that a small molecule called 1,4-benzodiazepines sensitized both resistant and tolerant strains of *C. albicans* to fluconazole (Alabi et al., 2023). Identifying adjuvant drugs which can enhance the efficacy of azoles represents a promising new avenue for discovering combination therapies. This approach offers a potential rapid adoption compared to the time needed for developing entirely new antifungal drugs.

## Conclusions

Although resistance to MCZ is relatively rare, this study indicates trisomy of ChrR mediated gain of tolerance to MCZ is the predominant strategy of adaptation to MCZ treatment in *C. albicans*. Further studies are required to investigate clinical relevance of MCZ tolerance. Hsp90 and calcineurin are essential for maintenance and development of MCZ tolerance. It will be interesting to perform screening for novel chemicals that inhibit Hsp90 or calcineurin, and then test if combination of the candidate chemical and MCZ can prevent happening of tolerance and resistance. Our findings underscore the need to assess the prevalence of MCZ tolerance in clinical isolates isolated before and after treatment with azoles. Identifying unique traits of tolerant strains will likely uncover new antifungal targets.

## Data availability statement

The sequencing data are available in the ArrayExpress database at EMBL-EBI (www.ebi.ac.uk/arrayexpress) under accession number E-MTAB-13880 and E557 MTAB-14133.

## Author contributions

YX: Conceptualization, Funding acquisition, Project administration, Supervision, Writing – original draft, Writing – review & editing. LZ: Formal analysis, Investigation, Methodology, Validation, Writing – review & editing. YD: Formal analysis, Investigation, Methodology, Validation, Writing – review & editing. CW: Investigation, Validation, Writing – review & editing. HD: Investigation, Validation, Writing – review & editing. ZW: Formal analysis, Investigation, Writing – review & editing. LG: Conceptualization, Funding acquisition, Supervision, Writing – review & editing.
