# Supplementary figures and images for: Miconazole induces aneuploidy-mediated tolerance in Candida albicans that is dependent on Hsp90 and calcineurin
Source: Front Cell Infect Microbiol. 2024 Jun 25;14:1392564. doi: 10.3389/fcimb.2024.1392564 (PMC11231705; doi:10.3389/fcimb.2024.1392564)

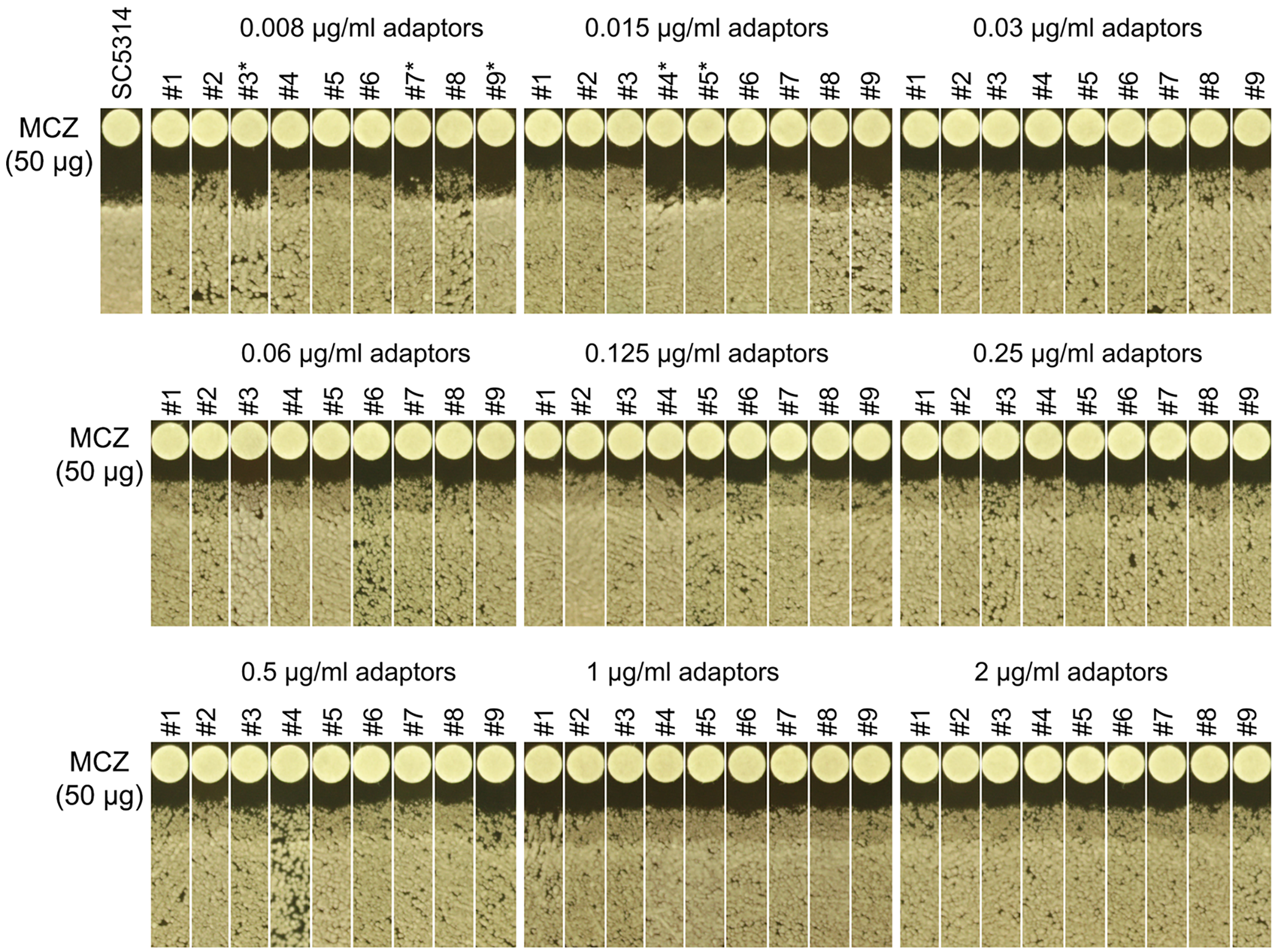

Supplement: Supplementary file 1 [file Image_1.tif]

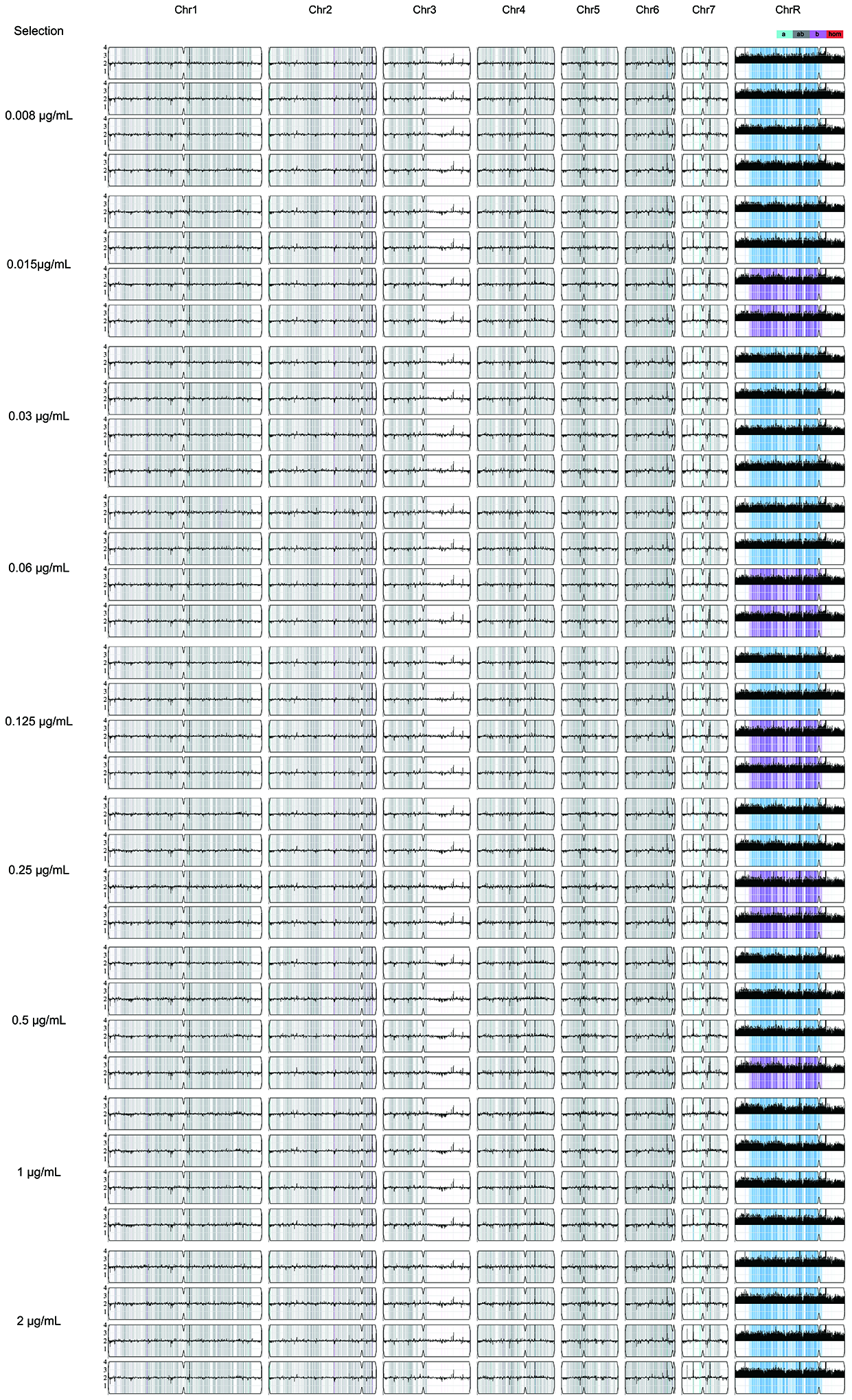

Supplement: Supplementary file 2 [file Image_2.tif]

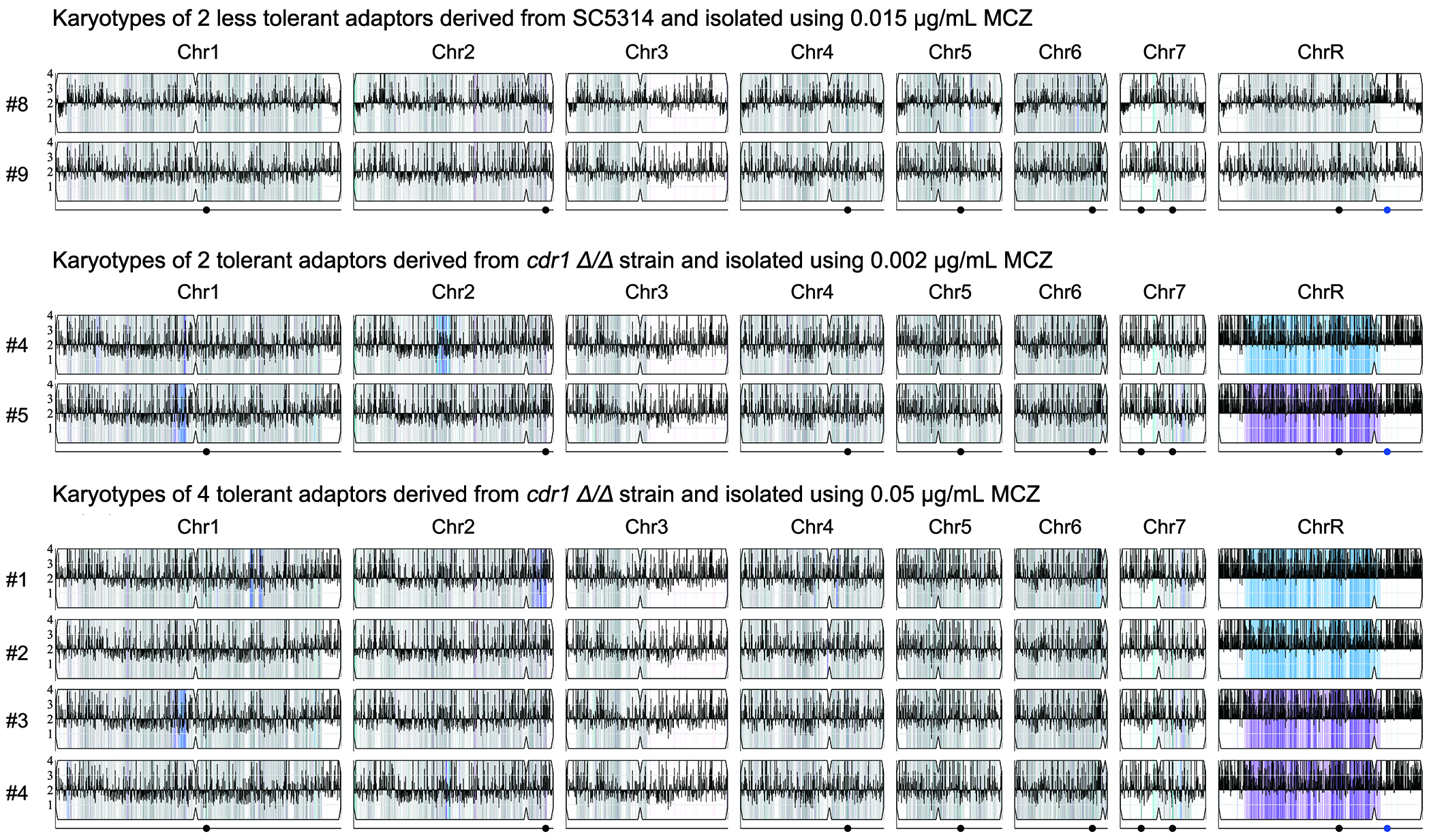

Supplement: Supplementary file 3 [file Image_3.tif]

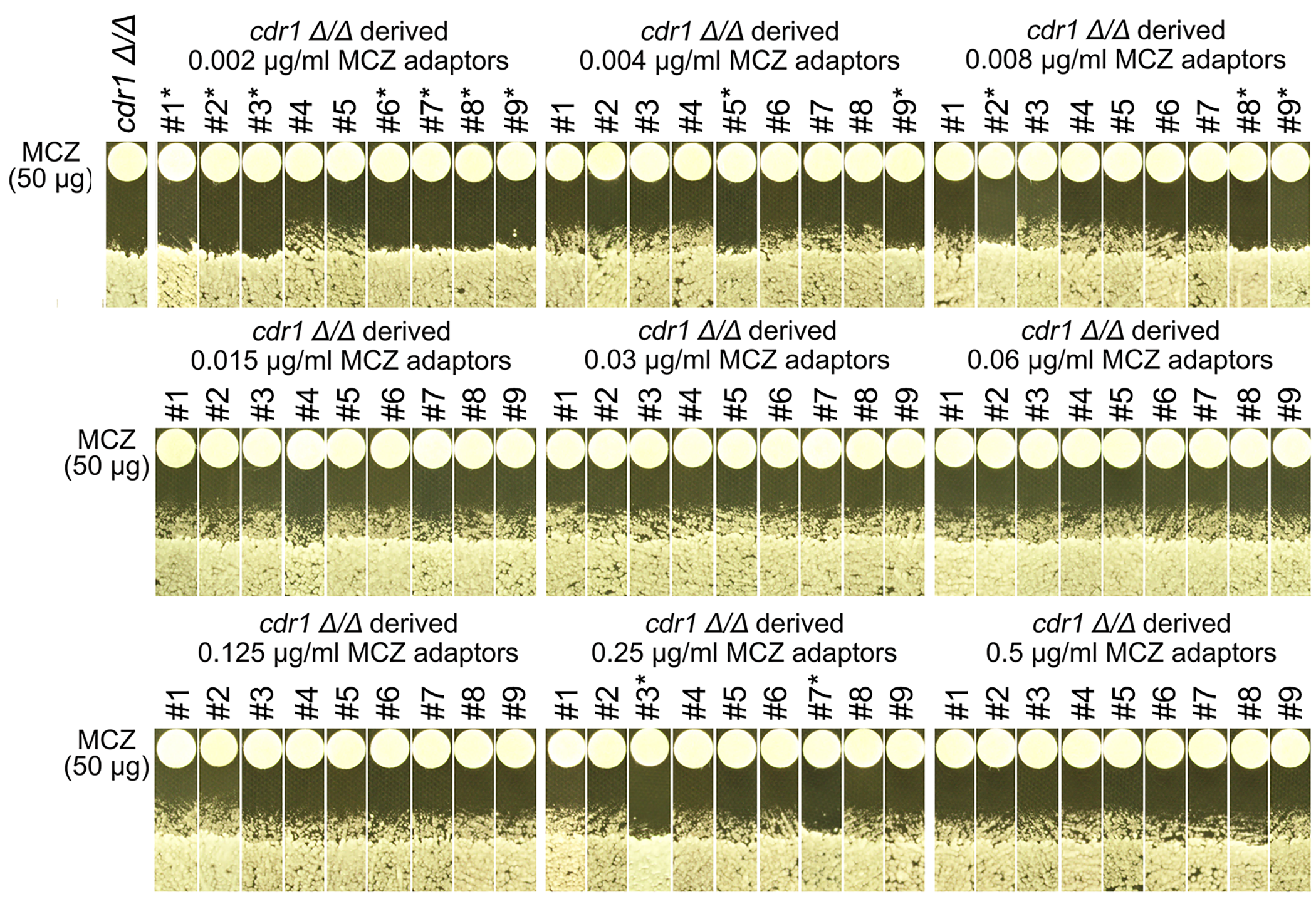

Supplement: Supplementary file 4 [file Image_4.tif]

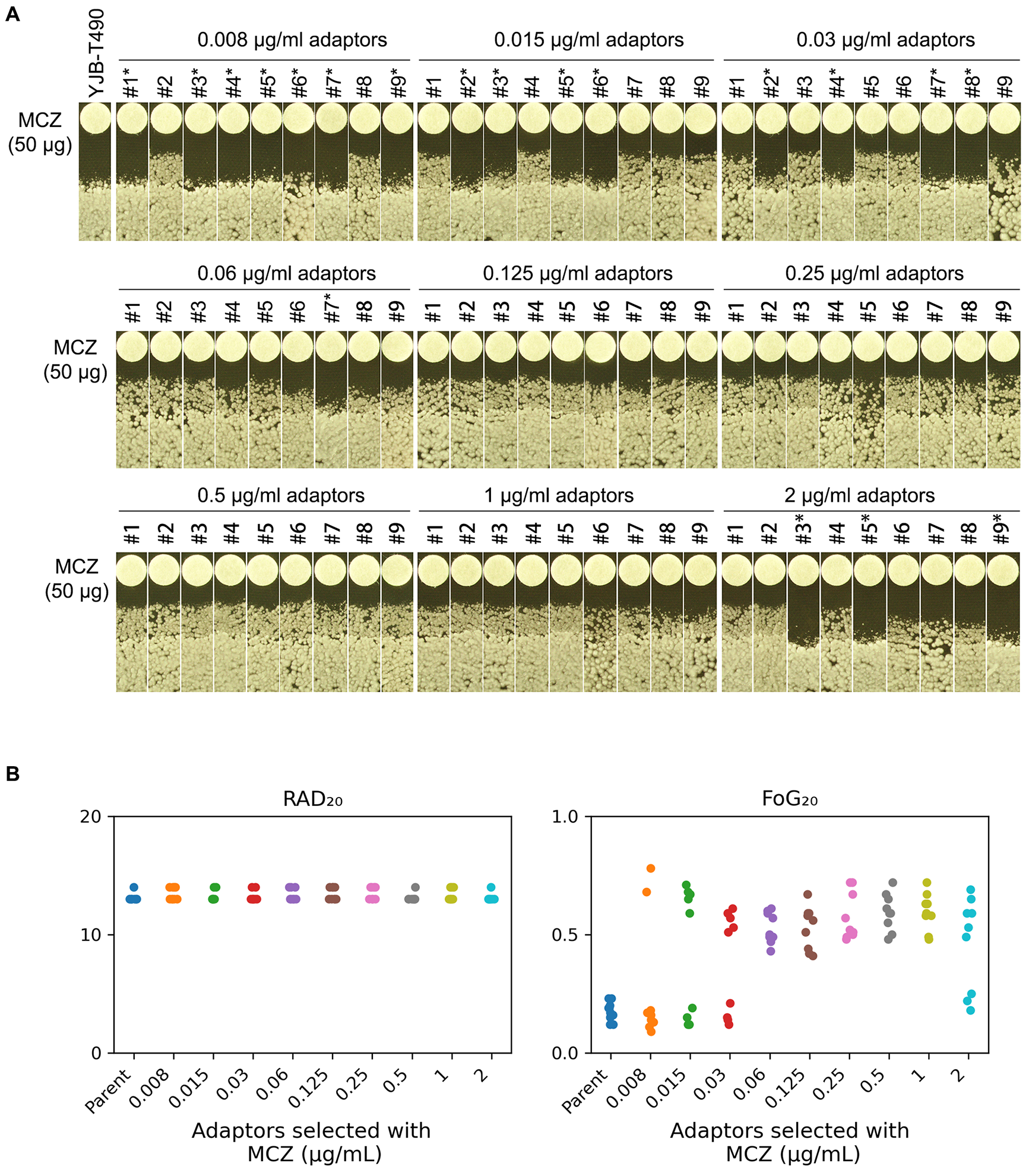

Supplement: Supplementary file 5 [file Image_5.tif]

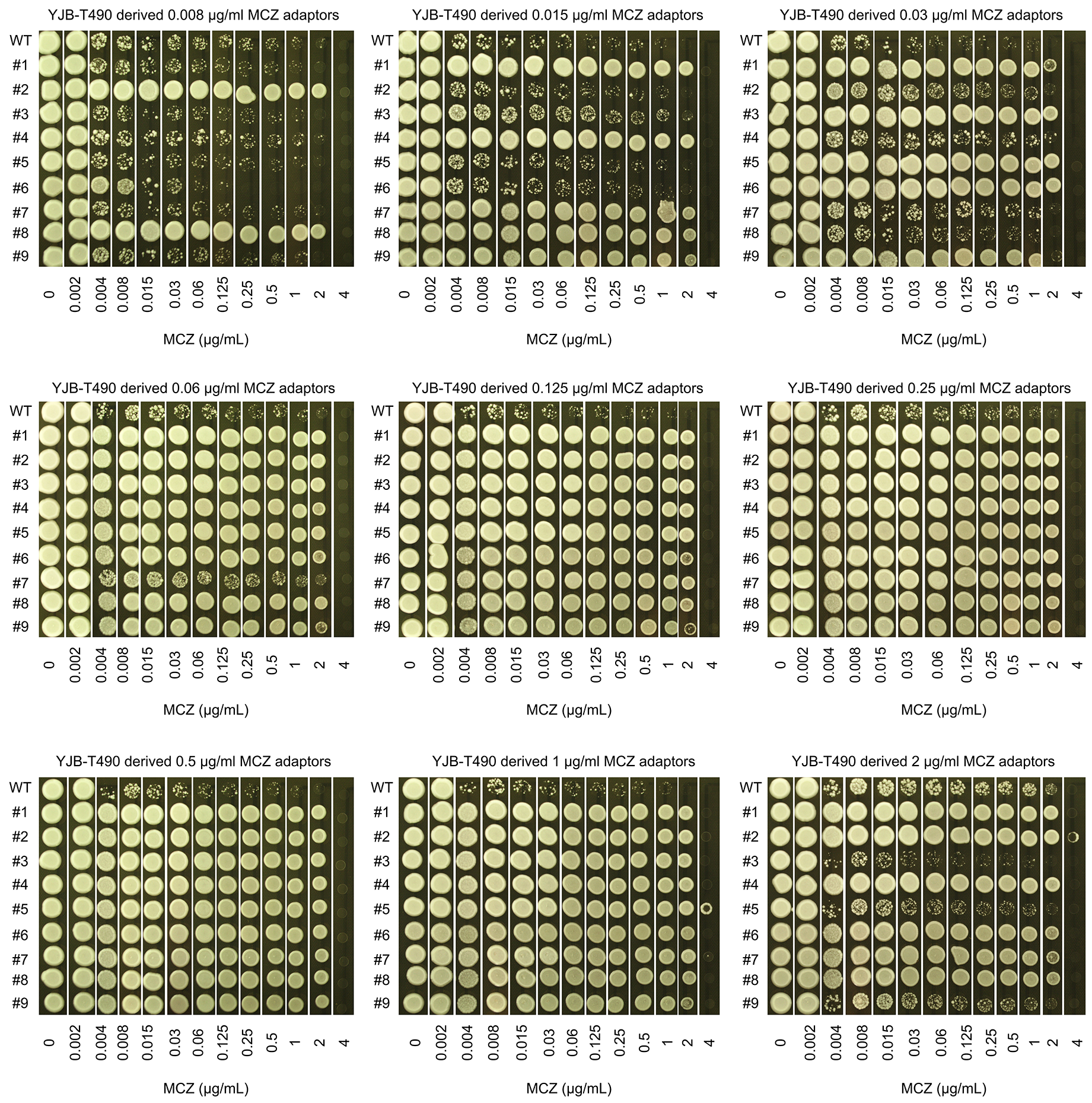

Supplement: Supplementary file 6 [file Image_6.tif]

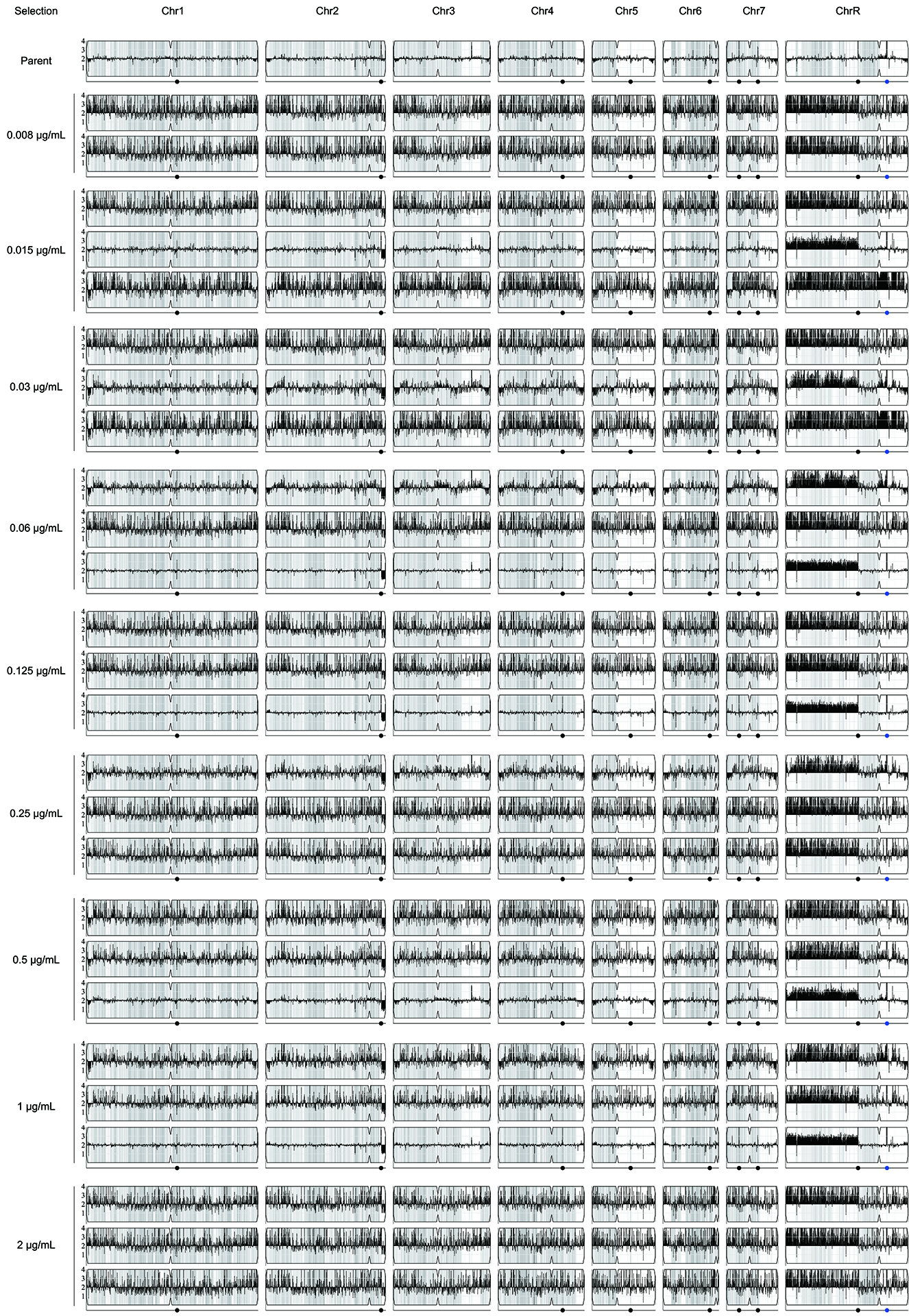

Supplement: Supplementary file 7 [file Image_7.tif]
