# Supplementary material for: Miconazole induces aneuploidy-mediated tolerance in Candida albicans that is dependent on Hsp90 and calcineurin
Source: Front Cell Infect Microbiol. 2024 Jun 25;14:1392564. doi: 10.3389/fcimb.2024.1392564 (PMC11231705; doi:10.3389/fcimb.2024.1392564)
Supplement: Supplementary file 8 [file Table_1.docx]

**Table S1. Strains used in this study**

| Strain | Genotype | Parent | Source |
| --- | --- | --- | --- |
| SC5314 | Wild type |  | [1] |
| YJB-T1891 | Wild type |  | [2] |
| YJB-T490 | Wild type |  | [2] |
| YCA779 | cdr1::FRT/cdr1::NAT1 flp | SC5314 | [3] |
| YCA776 | cdr2::FRT/cdr2::NAT1 flp | SC5314 | [3] |
| YCA652 | mdr1::FRT/mdr1::NAT1 flp | SC5314 | [3] |
| YCA641 | cmp1::FRT/cmp1::NAT1 flp | SC5314 | [3] |
| YCA623 | cnb1::FRT/cnb1::NAT1 flp | SC5314 | [3] |
| YCA736 | crz1::FRT/crz1::NAT1 flp | SC5314 | [3] |
| T1483 | tac1::FRT/tac1::NAT1 flp | SC5314 | This study |

**References**

1. Nobile, C.J. and A.D. Johnson, *Candida albicans Biofilms and Human Disease.* Annu Rev Microbiol, 2015. **69**: p. 71-92.

2. Yang, F., et al., *Antifungal Tolerance and Resistance Emerge at Distinct Drug Concentrations and Rely upon Different Aneuploid Chromosomes.* mBio, 2023. **14**(2): p. e0022723.

3. Xu, Y., et al., *Multifactorial Mechanisms of Tolerance to Ketoconazole in Candida albicans.* Microbiol Spectr, 2021. **9**(1): p. e0032121.
