# Supplementary material for: Miconazole induces aneuploidy-mediated tolerance in Candida albicans that is dependent on Hsp90 and calcineurin
Source: Front Cell Infect Microbiol. 2024 Jun 25;14:1392564. doi: 10.3389/fcimb.2024.1392564 (PMC11231705; doi:10.3389/fcimb.2024.1392564)
Supplement: Supplementary file 9 [file Table_2.docx]

**Table S2. Sequences of primers used in this study**

| Primer name | Primer sequence (5' to 3') |
| --- | --- |
| Gene deletions | |
| CaTAC1-US-F | GGGACGCAGTACATATAATAAAG |
| NAT1-CaTAC1-US-R | GTATAGGAACTTCCTCGAGGGGAGAACAACAGAATAGAGAGGG |
| NAT1-CaTAC1-DS-F | AGATCCACTAGTTCTAGAGCGGGAGTTGTAATTGGTGAAAGCG |
| CaTAC1-DS-R | GGGTATATGAATATAAAGTCTCGG |
| Diagnostic PCR for deletions | |
| CaTAC1-USD-F | CCTTCTTTTAGCGCTTCC |
| CaTAC1-DSD-R | CCATAATGTATCCAATTTCCG |
