# Supplementary material for: Miconazole induces aneuploidy-mediated tolerance in Candida albicans that is dependent on Hsp90 and calcineurin
Source: Front Cell Infect Microbiol. 2024 Jun 25;14:1392564. doi: 10.3389/fcimb.2024.1392564 (PMC11231705; doi:10.3389/fcimb.2024.1392564)
Supplement: Supplementary file 10 [file Table_3.docx]

# Supplementary materials

**Table S1. Strains used in this study**

**Table S2. Sequences of primers used in this study.**

**Figure S1. Disk diffusion assay of SC5314 derived miconazole adaptors**

Randomly, 9 colonies (adaptors) from each of the drug plates were tested with disk diffusion assays. The disks contained 50 μg miconazole. The plates were incubated at 30°C for 2 days then photographed. * indicates non-tolerant adaptors.

**Figure S2. Karyotypes of SC5314 derived tolerant adaptors**

Approximately one million cells of SC5314 were spread on YPD-agar plates supplemented with 0.008-2 μg/mL MCZ. From each plate, randomly 4 tolerant adaptors were sequenced. Shown are the sources and karyotypes of these adaptors.

**Figure S3. Karyotypes of SC5314 derived less tolerant adaptors and *CDR1* deletion strain derived tolerant adaptors**

A. Two adaptors (#8 and #9) derived from SC5314 and isolated from 0.015 μg/mL MCZ plate, were less tolerant to MCZ. They were sequenced and the karyotypes were shown.

B. Some tolerant adaptors derived from *CDR1* deletion strain were sequenced. Shown are karyotypes of 2 adaptors (#4 and #5) isolated from 0.002 μg/mL MCZ plate, and 4 adaptors (#1-#4) isolated from 0.5 μg/mL MCZ plate.

**Figure S4. Disk diffusion assay analysis of *cdr1 Δ/Δ* derived miconazole adaptors**

81 adaptors derived *cdr1 Δ/Δ* strain were tested with disk diffusion assays. The disks contained 50 μg miconazole. The plates were incubated at 30°C for 2 days then photographed. * indicates non-tolerant adaptors.

**Figure S5. Disk diffusion assay analysis of YJB-T490 derived adaptors**

A) Approximately one million cells of SC5314 were spread on YPD-agar plates supplemented with 0.008-2 μg/mL MCZ. From each plate, randomly 9 adaptors were tested with disk diffusion assays. Each disk contained 50 μg MCZ. Source of the adaptors are indicated in the figure. The plates were incubated at 30°C for 2 days then photographed. B) Plates of disk diffusion assays were analyzed using diskImageR. Shown are the RAD20 and FoG20 values. Sources of the adaptors are indicated in the figure. For the parent YJB-T490, 10 individual colonies were tested as 10 biological replicates

**Figure S6. Spot assay analysis of YJB-T490 derived adaptors.**

The same adaptors descried in Figure S3 were tested with spot assays using YPD-agar plates supplemented with various concentrations of MCZ. Shown are the source of the adaptors, as well as concentrations of MCZ used for spot assays. The plates were incubated at 30°C for 2 days then photographed.

**Figure S7. Karyotypes of YJB-T490 derived tolerant adaptors**

Some tolerant adaptors derived from YJB-T490 were sequenced. The sources of the adaptors are indicated in the figure. The karyotype of the wild type strain is also shown in the figure. Black dots indicate the loci of MRS on each chromosome. Blue dots indicate the loci of rDNA on chromosome R.
